# Supplementary material for: Experimental and Theoretical Study on the Synergistic Inhibition Effect of Pyridine Derivatives and Sulfur-Containing Compounds on the Corrosion of Carbon Steel in CO2-Saturated 3.5 wt.% NaCl Solution
Source: Molecules. 2018 Dec 11;23(12):3270. doi: 10.3390/molecules23123270 (PMC6321393; doi:10.3390/molecules23123270)
Supplement: Supplementary file 1 [file molecules-23-03270-s001.zip › Supplementary data.docx]

**Supplementary data of “Experimental and theoretical study on the synergistic inhibition effect of pyridine derivatives and sulfur-containing compounds on the corrosion of carbon steel in CO2-saturated 3.5 wt.% NaCl solution”**

**







Figure S1.** The curve of linear polarization under a potential range from –10 mV to +10 mV (vs. OCP) at sweep rate of 0.2 mV s^-1^ at 10 minutes interval: (a) blank, (b) adding mixture inhibitors contained 4-PQ and TU at the beginning, (c) adding 4-PQ at beginning, then adding TU at the time of 40th minutes, (d) adding TU at beginning, then adding 4-PQ at the time of 40th minutes.

**Table S1.** The polarization resistance calculated by linear polarization curve fitting.

| Time (min) | R_p_ (Ω∙cm^2^) | | | |
| --- | --- | --- | --- | --- |
|  | (a) | (b) | (c) | (d) |
| 5 | 135.58 | 757.6 | 423.47 | 215.16 |
| 10 | 131.64 | 763.9 | 455 | 255.58 |
| 15 | 139.47 | 745.06 | 453.09 | 279.79 |
| 20 | 151.5 | 742.52 | 458.41 | 280.56 |
| 25 | 148.29 | 748.56 | 564.69 | 494.49 |
| 30 | 157.31 | 773.79 | 610.34 | 561.52 |
| 35 | 152.08 | 777.88 | 695.44 | 585.31 |
| 40 | 155.76 | 785.9 | 740.66 | 610.62 |
